# Additional Facts and Observations Relative to Opium Applied Externally, so as to Be Absorbed by the Lymphatics

**Published:** 1799-08

**Authors:** M. Ward

**Affiliations:** Surgeon to the Manchester Infirmary


FOR THE MEDICAL AND PHYSICAL JOURNAL.

### *Additional Facts and Obfervations relative to Opium applied externally, fo as to be abforbed by the Lymphatics.—By* M. WARD, *Surgeon to the Manchefter Infirmary.*

HAVING endeavoured to excite the attention of medical practitioners to this interefting fubject*, it appears to be a duty incumbent upon me, to communicate any farther cafes or obfervations which may have occurred to me, tending either to elucidate or extend the practice.

The following, I truft, will not be deemed entirely unworthy their attention.

June 7, 1799, John Jackfon, at 53, was admitted into the Infirmary, with a fimple fracture of the tibia and fibula of the left leg. An eighteen-tailed bandage, moiftened in aq. litharg. acetat. comp. was applied, and the limb was

* See the Medical and Physical Journal for July.

was placed on its outer fide, with the knee bent. He was unufually loquacious whilft we were reducing the fracture. A low diet and a laxative medicine were prefcribed.

June 11. He has had very little fleep laft night, and was found this morning in a maniacal ftate, lying acrofs the bed without any of the bandages upon his leg. He talks incoherently: his tongue is white, and his countenance flufhed: pulfe 108 and foft. Circular rollers fteeped in aq. lith. acet. co. were applied upon the limb; and as foon as a ftrait waiftcoat could be procured, he was placed on his back, and his leg was confined in a fracture box, of fuch a conftruction as to allow his knee to be in a bent pofition, and the limb to be raifed. His leg was fecured in the box, and the potus acid. vegetab. directed to be drank ad libitum.

June 12. He found means in the night to extricate himfelf from the ftrait waiftcoat and has been fo noify the two laft nights, as to difturb the patients in the neighbouring wards: he never ceafes talking; generally mutters to himfelf; but fometimes is extremely noify: his eyes are blood-fhot, a conftant tremulous motion prevails in every part of his frame; his tongue and gums are much furred (the former has brown ftreaks upon it); pulfe 120.

R/. Amon. præp. gr. xv; pulv. cort. Peruv. fcrup. i; tinct. opii gutt. v. fyrup. facchar. drachm i; aq. cinam. drachm ii; aq. pur. drachm vi.—M. f. hauft. quarta quaque horâ fumend. in effervefcent. cum fuc. limon. recent. unc. fs.—Contin. potus.

I vifited him again at nine in the evening. He had taken two draughts. The tremor and delirium had not abated; I therefore directed the following liniment to be rubbed into the infide of his right thigh immediately, and to be repeated in four hours, unlefs fleep was procured:

R/. Opii pulv. fubtil. drachm fs. camphor: gr. iv. adip. fuil: fcrup. iv. ol. olivar. drachm i. M.—*

June 13. He flept well the whole of the laft night, the tremor is gone and he is eafy, compofed and rational: his thirft is abated; but his tongue continues white: appetite good, pulfe 88.

The ftrait waiftcoat was taken off: he has no complaint left, except a flight mazinefs in his head. Two portions of liniment, each containing half a drachm of opium, were applied.

He has been haraffed with a great variety of incongruous ideas fince the delirium came on; but that which feems to have made the ftrongeft impreffion is, his imagining himfelf to have been conveyed with incredible velocity, from

* This formula seems to be absorbed with more ease than any other I have yet tried but one drachm of lard and half a drachm of oil is sufficient, where the opium does not exceed a scruple, and so in proportion.

from one eminence to another. It will be unneceffary to continue the journal regularly. It did not feem fafe at once to difcontinue the opium: one portion of liniment was therefore rubbed in every night for three or four times; but each portion contained a fmaller quantity of opium than the preceding.

On the 14th, in the morning, his pulfe was 92 and regular; in the evening 80, 15th 84, 16th 76, 17th 84, 19th 80. Before the firft portion of opium was applied, his pulfe fluctuated between 100 and 120. July 4. His leg is as ftraight and as firm for the time, (28 days) as any fractured limb I ever faw, which is furprifing, confidering how reftlefs he has been.

The mania has returned at intervals fince the 20th ult. and he has taken two grains of opium every night lately, with apparent advantage; but has often been confined fince the 20th, by the waiftcoat in the night, and liberated in the day time. His appetite has been infatiable fince the 18th. ult. Only one perfon has vifited him fince his admiffion, and fhe is ignorant of his previous hiftory. He fays he never was infane before.

To guard againft the indifcriminate application of opium externally, by abforption, in delirium accompanied with fever, it may be proper to obferve, that in a receut inftance of typhus, where petechiæ, a livid appearance of the efchar, occafioned by a blifter, ftupor, and an involuntary difcharge of bloody urine, had taken place, and the patient had been delirious and had had very little fleep for fome time, a fcruple of opium was rubbed into one thigh (15 grains had been rnbbed in fix hours before, and ten grains twelve hours before, without producing any fenfible effect). The next day the patient was affected with coma, which went off in about 24 hours.

The ftupor and putridity which prevailed in the fyftem before the opium was employed, were propably amply fufficient to account fatisfactorily for the occurrence of this fymptom; but ftill, the external application of opium, except in fmall quantities, when there is any tendency to coma, feems likely to be ambiguous, if not hurtful in its effects.

Mary Caldwell did not feem to derive any material advantage from a continuance of frictions with opium; they were therefore difcontinued; but they enabled her to leave off taking the pulv. ipecac. comp. which could not be accomplifhed till fhe had recourfe to them. Since that time fhe is become an home patient to the Infirmary, and was no better the laft time I heard of her.

Dr. Percival informs me, that fince his laft communication to me, he has tried the external ufe of opium in a chronic dyfury, and in a cafe of the ftone in the bladder, with very confiderable eafe to the patients, and without producing the vertigo, head-ach, and obftinate coftivenefs, which the internal ufe of laudanum had before occafioned. He has fuggefted to me the following experimental inquiries.

1. What

1. What is the smallest quantity of unguent required for combination with the opium, so as to render it readily admissible into the body? To ascertain this point, might tend to facilitate and shorten the operation of inunction.

2. Would the oleum e pedibus bovinis, or neats' foot oil, which, being remarkably lubricating, may be supposed to pass readily into the pores of the skin, be a commodious vehicle for the opium?

3. Would opium, combined with the yolk of an egg, gain a readier admission into the body, than with an oily substance?

*** In a part of the impression, page 7, of this Number, several words have accidentally escaped correction; the reader is therefore requested to correct the *Note*, as follows:—after the word, "but," for "—— of lard"" read, "*a drachm of lard;*" and for "—— of oil, read, "*half a drachm of oil.*"

